# Issue Information

**DOI:** 10.1002/cam4.148

**Published:** 2013-10-02

**Aims and Scope**

*Cancer Medicine* is a peer-reviewed, open access, interdisciplinary journal providing rapid publication of cutting-edge research from global biomedical researchers across the cancer sciences. The journal considers submissions from all oncologic specialties, including, but not limited to, the following areas:

**Cancer biology**

Molecular biology •cellular biology •molecular genetics •genomics •immunology •epigenetics •metabolic studies •proteomics •cytopathology •carcinogenesis •drug discovery and delivery

**Clinical cancer research**

Translational research •clinical trials •chemotherapy •radiation therapy •surgical therapy •clinical observations •clinical guidelines •genetic consultation •ethical considerations

**Cancer prevention**

Behavioral science •psychosocial studies •screening •nutrition •epidemiology and prevention •community outreach

*Cancer Medicine* publishes original research articles, systematic reviews, meta-analyses, and research methods papers, along with invited editorials and commentaries. Original research papers must report well-conducted research with conclusions supported by the data presented in the paper.

We aim to be a truly global forum for high-quality cancer research, and we think that the best research should be published and made widely accessible as quickly as possible. *Cancer Medicine* publishes papers submitted directly to the journal and those referred from a select group of prestigious journals published by Wiley-Blackwell.

*Cancer Medicine* is a Wiley Open Access journal, one of a new series of peer-reviewed titles publishing quality research with speed and effi ciency For further information visit the Wiley Open Access website at http://www.wileyopenaccess.com.

**Open Access and Copyright**

All articles published by *Cancer Medicine* are fully open access: immediately freely available to read, download and share. All articles accepted from 14 August 2012 are published under the terms of the Creative Commons Attribution License. All articles accepted before this date were published under a Creative Commons Attribution Non-Commercial License. The Creative Commons Attribution License permits use, distribution and reproduction in any medium, provided the original work is properly cited and allows the commercial use of published articles. Copyright on any research article in a journal published by *Cancer Medicine* is retained by the author(s). Authors grant Wiley a license to publish the article and identify itself as the original publisher. Authors also grant any third party the right to use the article freely as long as its integrity is maintained and its original authors, citation details and publisher are identifi ed. Further information about open access license and copyright can be found at http://www.wileyopenaccess.com/details/content/12f25db4c87/Copyright--License.html.

**Purchasing Print Reprints**

Print reprints of Wiley Open Access articles can be purchased from corporatesales@wiley.com.

**Disclaimer**

The Publisher and Editors cannot be held responsible for errors or any consequences arising from the use of information contained in this journal; the views and opinions expressed do not necessarily refl ect those of the Publisher and Editors, neither does the publication of advertisements constitute any endorsements by the Publisher and Editors of the products advertised.

Wiley Open Access articles posted to repositories or websites are without warranty from Wiley of any kind, either express or implied, including, but not limited to, warranties of merchantability, fi tness for a particular purpose, or non-infringement. To the fullest extent permitted by law Wiley disclaims all liability for any loss or damage arising out of, or in connection with, the use of or inability to use the content.

**Editor-in-Chief**

Qingyi Wei, M.D., Ph.D. *The University of Texas MD Anderson Cancer Center, Houston, USA*

Gang Li

*University of British Columbia, Vancouver, Canada* Youyong Lu

*Beijing Institute for Cancer Research, Beijing, China* Kenneth Offi t

*Memorial Sloan-Kettering Cancer Center, New York, USA* Athanasios G. Papavassiliou *University of Athens Medical School, Athens, Greece*

Richard J. Ablin *University of Arizona College of Medicine, Tucson, USA* Rosalind Eeles *Institute of Cancer Research, Sutton, UK*

Elizabeth A. Grimm *The University of Texas MD Anderson Cancer Center, Houston, USA* Giseli Klassen *Federal University of Parana, Curitiba, Brazil*

**Editorial Board Cancer Biology**

Kenneth Pienta

University of Michigan, An Arbor,

USA

Jack A. Schalken

Radboud University Medical Centre,

Nijmegen, Netherlands

Kazu Ushijima

National Cancer Center, Tokyo, Japan

David Vaux

Walter and Eliza Hall Institute

of Medical Research, Parkville,

Australia

Cun-Yu Wang *UCLA School of Dentistry, Los Angeles, USA* Hongyang Wang *Eastern Hepatobiliary Surgery Institute / Hospital, Shanghai, China* Momiao Xiong *The University of Texas Health Science Center, Houston, USA* Li Zhang

The University of Texas MD Anderson Cancer Center, Houston, USA

**Clinical Cancer Research**

Yung-Jue Bang

Seoul National University College

of Medicine, Seoul, Korea

Anthony Chan

The Chinese University of Hong

Kong, China

Peter J. Fuller

Prince Henry Institute, Victoria,

Australia

Maura L. Gillison

Ohio State University

Comprehensive Cancer Center,

Columbus, USA

Guido Kroemer

University of Paris Descartes, Paris,

France

Lalit Kumar

All India Institute of Medical

Sciences, New Delhi, India

Razelle Kurzrock

The University of Texas MD

Anderson Cancer Center, Houston,

USA

Geoffrey Liu

Princess Margaret Hospital, Toronto,

Canada

Jiade J. Lu

National University of Singapore,

Singapore

Li Mao

University of Maryland, Baltimore,

USA

Akira Nakagawara

Chiba University, Chiba, Japan

Pamela L. Paris

University of California,

San Francisco, USA

Dimitrios H. Roukos

Ioannina University School of

Medicine, Ioannina, Greece

Frank A. Sinicrope

Mayo clinic, Rochester, USA

Jun Wang

BGI-Shenzhen, Shenzhen, China

Glen J. Weiss

Translational Genomics Research

Institute, Phoenix, USA

Eric T. Wong

Beth Israel Deaconess Medical

Center, Boston, USA

**Cancer Prevention**

Marianne Berwick *University of New Mexico, Albuquerque, USA* Paolo Boffetta *The Tisch Cancer Institute, New York, USA* Eduardo Cazap *Latin-American & Caribbean Society of Medical Oncology, Buenos Aires, Argentina* Brock C. Christensen *Dartmouth Medical School, Hanover, USA* Steve Dubinett *David Geffen School of Medicine at UCLA, Los Angeles, USA*

Kari Hemminki

German Cancer Research Center,

Heidelberg, Germany

Zhibin Hu

Nanjing Medical University,

Nanjing, China

Stephen D. Hursting

The University of Texas, Austin,

USA

Daehee Kang

Seoul National University College

of Medicine, Seoul, Republic of

Korea

Lambertus A. Kiemeney

Radboud University Medical

Centre, Nijmegen, Netherlands

Dongxin Lin

*Chinese Academy of Medical Sciences, Beijing, China* Somdat Mahabir *National Cancer Institute, Bethesda, USA* Girish Maru

*ACTREC Tata Memorial Centre, Navi Mumbai, India* Andrew Olshan *University of North Carolina, Chapel Hill, USA* Jong Y. Park

H. Lee Moffi tt Cancer Center & Research Institute, Tampa, USA

Susan K. Peterson

The University of Texas MD

Anderson Cancer Center, Houston,

USA

Thomas E. Rohan

Albert Einstein College of

Medicine, Bronx, USA

Xifeng Wu

The University of Texas MD

Anderson Cancer Center,

Houston, USA

**Editorial Advisory Board**

Lawrence Baker

University of Michigan, Ann Arbor,

USA

David Christiani

Harvard School of Public Health,

Boston, USA

Robert B. Diasio

Mayo Clinic, Rochester, USA

B. Mark Evers

Lucille P. Markey Cancer Center,

Lexington, USA

Waun Ki Hong

The Univeristy of

Texas MD Anderson

Cancer Center, Houston,

USA

Ernest Hawk *The University of Texas MD Anderson Cancer Center, Houston, USA*

Leroy Hood

Institute for Systems Biology, Seattle, USA

James Mulé

H. Lee Moffi tt Cancer Center &

Research Institute, Tampa, USA

Philip Agop Philip

Wayne State University, Detroit, USA

Nicholas Vogelzang

Comprehensive Cancer Centers of

Nevada, Vegas, USA

